# Segmental Trunk Control in Sitting Between Full-Term and Preterm Infants Raised in the Orphanage Setting

**DOI:** 10.3390/ijerph22121824

**Published:** 2025-12-05

**Authors:** Nurul Fauziah Arifin, Wantana Siritaratiwat, Ponlapat Yonglitthipagon, Lugkana Mato, Thiwaphon Chatprem, Noppharath Sangkarit

**Affiliations:** 1 Research Center in Back, Neck, Other Joint Pain and Human Performance (BNOJPH), Faculty of Associated Medical Sciences, Khon Kaen University, Muang, Khon Kaen 40002, Thailand; nurulfauziah.a@kkumail.com (N.F.A.); thiwch@kku.ac.th (T.C.); 2 School of Physical Therapy, Faculty of Associated Medical Sciences, Khon Kaen University, Muang, Khon Kaen 40002, Thailand; ponlapat@kku.ac.th (P.Y.); yui@kku.ac.th (L.M.); 3 Department of Physical Therapy, School of Allied Health Sciences, University of Phayao, Phayao 56000, Thailand; noppharath.sa@up.ac.th

**Keywords:** environmental contexts, caregiver interaction, segmental trunk control, orphanage setting, child-rearing practice

## Abstract

**Background**: Postural control is vital for independent sitting in infants. Those in orphanages face environmental limitations and biological factors that affect trunk control. This study aimed to compare segmental trunk control between full-term and preterm/low-birth-weight infants and examine the correlations with daily activities. **Methods**: Thirty-three infants (16 full-term, 17 preterm/low birth weight) were assessed using the Segmental Assessment of Trunk Control (SATCo), and 27 were observed with the Daily Activity of Infant Scale (DAIS) over three weekdays and two weekends. **Results**: A slight significant difference (*p* < 0.05) in SATCo scores was found, with full-term infants scoring higher. Results found that both prenatal exposure and environmental factors could influence trunk control. A significant correlation (r_s_ = 0.771, *p* < 0.001) between trunk control and daily activities underscores the importance of upright and antigravity activities for postural development. A small-to-medium effect size suggested limited practical significance due to being conducted in a single orphanage with a homogeneous sample. **Conclusions**: The findings emphasize enhancing the orphanage environment and child-rearing practices to support trunk control development through upright posture, movement exploration, and high-quality caregiver interactions.

## 1. Introduction

Postural control is vital in developing gross motor skills throughout early childhood, particularly in achieving and maintaining independent sitting [1,2,3]. Attaining independent sitting is a significant gross motor milestone during the first year of life [4]. Research indicates that learning to sit is a critical period for infants to engage with their environmental context, including child-rearing and daily activities [5]. Additionally, postural control also directly affects milestones such as crawling and walking, allowing infants to maintain stability and shift weight, which aids their transition from horizontal to vertical movement during daily activities [6].

Environmental contexts, such as movement experiences, child-rearing practices, and interactions with mothers or caregivers, are essential factors that can impact postural control during sitting [7,8,9,10]. It was found that child-rearing culture and prior sitting experiences enhance independent sitting skills by the age of five months [9]. It is also suggest that the opportunity to sit unsupported from around four months, along with experiencing sitting on various surfaces during the child-rearing period, may augment segmental trunk control in moderate-to-late preterm infants [11].

Children residing in orphanages experience different environmental contexts compared to those raised by their families. The level of physical restriction varies among orphanages, and other contextual factors (e.g., caregiver-to-infant ratio) could influence motor development [5,12]. Being raised in orphanages, infants typically spend a significant amount of their time in confined spaces and frequently have limited opportunities for movement and exploration. Furthermore, orphaned infants are restricted in their free play and interaction within their surroundings [13,14]. Insufficient caregiver interaction focuses solely on basic needs, hindering active play and the exchange of feedback. This lack of interactive exploration during routines is commonly linked to delays in motor development [5,12].

Apart from limited environmental contexts, infants in orphanages also face increasing biological risks, especially those born to mothers with substance use, maternal mental illness, premature birth, and low birth weight [15,16,17,18]. Previous study highlighted that orphanage infants tend to exhibit lower variability in gross motor skills, particularly those with biological risk factors such as maternal psychological illness and premature birth [19]. Preterm infants exhibit less complex head control and fewer postural strategies, which increases the risk of delays in trunk control [20]. Low-birth-weight infants often experience hypotonia, making it challenging for them to maintain a stable posture, which further affects their ability to sit independently [21]. It is reported that only 10% of 20 late preterm infants achieved full trunk control by 6 to 8 months, whereas 70% of 36 full-term infants could sit independently by 8 months [22]. These delays could be worsened in orphanages, where opportunities for movement exploration and free play are limited.

To our knowledge, the measurement of segmental trunk control during a sitting posture in an orphanage setting is limited. This gap is notable because institutionalized infants are exposed to caregiving routines, environmental stimulation, and opportunities for movement that differ substantially from those typically experienced in family settings. These contextual factors, such as limited movement opportunities, limited upright positioning, and higher rates of biological and prenatal risks, may uniquely influence the development of trunk control. Therefore, investigating segmental trunk control specifically within an orphanage provides crucial scientific insight into how their trunk control develops and how this distinct environment contributes to early postural development.

This study thus aimed to examine segmental trunk control in orphaned infants and to compare segmental trunk control scores between full-term infants and those who are preterm or have a low birth weight. As their trunk control may be related to their daily activity, we secondarily examined the correlation between segmental trunk control and scores of observed routine daily activity. The findings will provide valuable insights into infants’ trunk control and activities, serving as guidelines for child-rearing practices in orphanages to promote better postural control of orphaned infants.

## 2. Materials and Methods

### 2.1. Study Design and Participants

Participants in this cross-sectional study were purposively recruited at an orphanage in Northeastern Thailand, involving two participant groups with a range of ages and/or corrected age of 3 to 8 months: full-term group and preterm and/or low-birth-weight group. This age range was selected because it is the golden period for attaining postural control in independent sitting [4]. Full-term infants were recruited as those who were born after 37 weeks of gestation, with a birth weight between 2500 and 4000 g and Apgar scores of 8 to 10. The preterm and/or low-birth-weight group included infants born before 37 weeks and/or with a birth weight below 2500 g. Infants were excluded if they displayed significant neurological abnormalities, muscle or joint deformities, visual or hearing deficits, a history of retinopathy of prematurity, or congenital anomalies. Before collecting the data, the researchers reviewed the infants’ medical records and history files. They inspected the infants’ muscles and joints, particularly those with suspected deformities, to ensure that their conditions did not meet the exclusion criteria.

A significance level of α < 0.05 and a statistical power (1 − β) of 80% (Z_0_._2_ = 0.84) were used to determine the sample size for comparing the difference in SATCo scores between two groups, calculations based on the mean difference (2.0) and standard deviation (3.5) from pilot data (5 infants in each group) required at least 17 infants in each group. To assess the correlation between segmental trunk control and daily activities, a correlation coefficient (r_s_ = 0.6) from our pilot study indicated the need for at least 27 infants.

### 2.2. Description of Child-Rearing in the Orphanage

The orphanage in this study accommodates about thirty infants aged 3 to 12 months on the second floor in a designated room. Their developmental progress varies; some have achieved standing and walking milestones, while others are still learning to sit, crawl, or cruise. Each infant has an individual cot with a pillow, a toy hanger, and a basket for milk bottles.

A team of six caregivers ensures consistent care through scheduled shifts on weekdays and weekends. Two to three caregivers are present in the mornings, including one focused on developmental stimulation. Two caregivers remain on duty in the evenings to attend to the infants’ needs.

Infants typically wake up between 4:00 and 5:00 a.m. for baths, dressing, and diaper changes. Milk is provided from 8:00 to 9:00 a.m., followed by independent playtime. Lunch is served around 11:00 a.m., followed by a period of calm rest. Afternoon milk is given around 2:30 p.m., and infants who can sit, crawl, stand, or learn to walk are allowed to play outside their cots. Dinner is usually fed at 4:30 p.m., and milk is provided between 8:00 and 10:00 p.m. for bedtime

### 2.3. Measurement Instruments

#### 2.3.1. Segmental Assessment of Trunk Control (SATCo)

The SATCo demonstrates strong inter-rater reliability and intra-rater reliability among both typically developing children and those with neurological conditions [23]. The assessment evaluates trunk control through seven components: head, upper thoracic, mid-thoracic, lower thoracic, upper lumbar, lower lumbar, and complete trunk control. Each component is assessed under static, active, and reactive control. Static control requires the individual to maintain a neutral posture at varying support levels, while active control involves sustaining posture during head movements. Reactive control tests the ability to maintain posture in the face of external perturbations.

**Figure 1:**
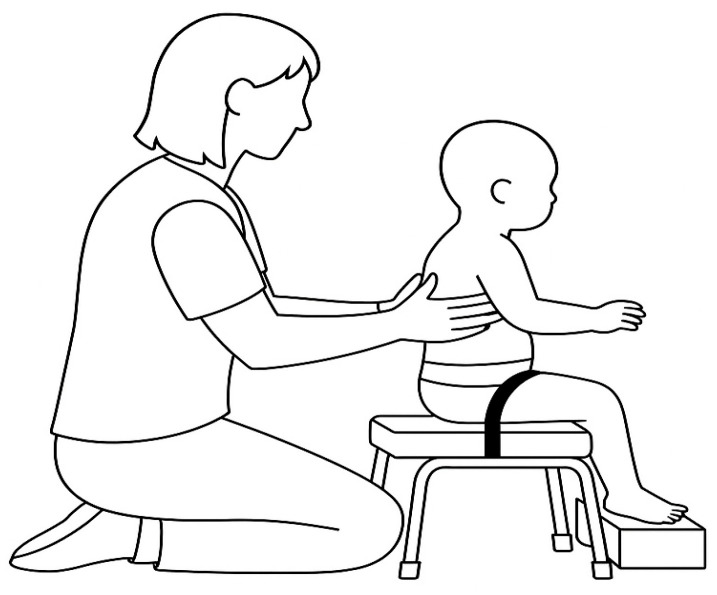
Infant position during SATCo test.

The SATCo was administered using the standardized procedures of Butler et al. [23]. Each assessment involved two assessors: one provided manual trunk support, and the other observed alignment, monitored arm and hand position, and applied the gentle perturbations required for reactive control. Infants wore a nappy or light clothing to allow clear visualization of trunk alignment. They were seated on a rigid bench with the pelvis and thighs stabilized using straps and the feet supported in neutral. The arms remained free and were not allowed to contact the examiner or bench, as detailed in Figure 1.

The manual support was provided at six segmental levels in a cephalocaudal sequence: axillae (upper thoracic), inferior angle of the scapula (mid thoracic), lower ribs (lower thoracic), below the ribs (upper lumbar), and pelvis (lower lumbar). At each level, the assessor placed both hands horizontally around the trunk, and had contact with only the lateral surfaces to permit the infant to generate postural control. Static, active, and reactive control were assessed at each segment. Static control was defined as maintaining a neutral upright posture for 5 s. Active control was assessed by the ability to turn the head ~45° to each side and return to midline without trunk collapse. Reactive control was evaluated by delivering brief, gentle nudges to the upper trunk and observing whether the infant returned to an upright position. Performance at each segment was recorded as present or absent. Control is recorded as 1 (present) or 0 (absent). If infants show segmental trunk control, SATCo scores range from 1 (head control) to 7 (complete trunk control). The maximum score is 7 in static and active conditions, and 6 in reactive conditions (head control is not evaluated). An “NT” score is assigned if the infant is unable to complete the test.

The intra-rater reliability test of SATCo involved five infants who could not sit independently and five who could, assessed one week apart at the orphanage. The researcher practiced the procedures for 2 months with an expert and received training from an orthopedic specialist on support levels. The intra-rater reliability of the SATCo was ICC _(3,1)_ = 0.968 (95% CI 0.877–0.992), while the inter-rater reliability between the researcher and an expert was ICC _(3,1)_ = 0.989 (95% CI 0.980–0.994).

#### 2.3.2. Daily Activity of Infants Scale (DAIS)

The Daily Activity of Infant Scale (DAIS, https://canchild.ca/wp-content/uploads/2025/03/DAIS2004.pdf?license=yes [Accessed on 15 May 2024]) is an instrument developed by Bartlett et al. designed to help parents or caregivers observe and recognize the exploration of children’s movement during their daily activities, thereby supporting the development of antigravity postural control. This instrument demonstrates strong inter-rater reliability, with an ICC range of 0.60–0.86, and reliable test–retest results, with an ICC range of 0.60–0.87 [5].

The DAIS contains eight dimensions of activity: feeding, bathing (including diaper changes), dressing, carrying, quiet play, active play, outings, and sleeping. It features 24 illustrated photographs that depict three levels of ability, as well as a separate image for sleep. Each dimension, except for sleep, is categorized into three ability levels: A, B, and C, where Level A indicates minimal opportunity for developing antigravity postural control and Level C denotes maximum opportunity.

Observers select the picture most closely representing the child’s activity level, marking a block for each 15 min observation. For example, if bathing occurs from 07:00 to 07:30, two blocks are marked at the corresponding ability level. Dimension scores are calculated by summing the marked blocks, with 1 point assigned to Level A, 2 points to Level B, and 3 points to Level C. Sleep is assigned a score of 0 because it does not contribute to active engagement in an upright posture position. Total scores are derived from all dimension scores. Within the same amount of time, the higher scores indicate greater opportunities for developing postural control and movement exploration.

### 2.4. Procedure

The study was conducted with the Declaration of Helsinki, and the protocol was approved by Khon Kaen University Ethics Committee for Human Research (Reference Number HE672077, Record No. 4.2.01:17/2567. Date of Approval: 18 June 2024). Written informed consent was obtained from the guardians of the orphans for their infants’ participation in the study. Researchers obtained permission from the orphanage’s Head to access each infant’s history file, which enabled us to objectively record the reasons for placement in the orphanage and the maternal conditions during pregnancy. The following characteristics of the orphaned infants were recorded: gender, gestational age, chronological age, corrected age, birth weight, birth height, head circumference, and Apgar scores at one and five minutes. The demographic data of preterm infants were discussed based on the infants’ corrected age, which corresponds to the chronological age of full-term infants. The age of admission to the orphanage, neonatal conditions, and the mother’s health during pregnancy (if available) were documented. Before conducting any tests, researchers measured the infants’ body temperature, weight, height, and head circumference to confirm that they were in good health.

The researchers assessed the segmental trunk control during sitting using the SATCo. Infants were supported at each level of their trunk, while the researcher monitored and scored the child’s posture across three conditions. Passing in each condition occurs when the infants maintain a neutral sitting posture. Failing happens if they cannot sustain an upright posture or respond to disturbances. The SATCo was administered to awake and attentive infants, utilizing stimuli such as distinct sounds and brightly colored toys to encourage movement. Trunk control was assessed once for each condition.

A researcher observed the daily routines of infants in the orphanage from 9:00 a.m. to 11:00 a.m. and from 1:00 p.m. to 4:00 p.m. Key activities observed included bathing, feeding, play, and sleeping, as well as the characteristics of the caregivers. Data collection occurred over three weekdays and two weekend days. The outing dimension was not assessed, as infants had no outdoor activities. Trunk control assessments and daily activity observations were conducted on a separate day due to the time required for thorough evaluation.

### 2.5. Data Analysis

Data analyses were conducted using SPSS for Windows version 28.0. Demographic data were analyzed with descriptive statistics. SATCo scores were reported numerically for trunk control in static, active, and reactive conditions, while DAIS scores reflected accumulated scores across dimensions. Due to the non-normal distribution of SATCo and DAIS scores, median and range were used for descriptive statistics. SATCo scores between full-term and preterm/low-birth-weight groups were compared using the Mann–Whitney U-test, with significance set at *p* < 0.05. The correlation between segmental trunk control and daily activities was also analyzed using the Spearman correlation coefficient (r_s_), with the following interpretations: r_s_ < 0.25 = little to no correlation, 0.25 < r_s_ < 0.50 = fair, 0.50 < r_s_ < 0.75 = moderate to good, and r_s_ > 0.75 = good to excellent [24].

## 3. Results

### 3.1. Demographic Data of Infants

The study recruited 33 infants, consisting of 16 full-term (8 females) and 17 preterm and/or low-birth-weight infants. As indicated in Table 1, the full-term group met the mean gestational age and birth weight criteria, whereas the preterm group was moderate to late preterm infants with slightly lower birth weights. At the time of data collection, all infants were in a healthy condition, had no fever, and had appropriate weight, height, and head circumference.

Infants were admitted to the orphanage because their families were unable to care for them due to poverty, parental imprisonment, or maternal mental health conditions. Table 2 shows that the highest prevalence of maternal conditions during pregnancy in the full-term group was the infants born to mothers with mental illness, and the highest maternal condition in the preterm and/or low-birth-weight group was the infants born to mothers who used drugs or alcohol.

Of the 33 infants, 11 could maintain a sitting position with and without support, while the remaining 22 could not. Two infants in each group, with a mean age of 8 months, could sit independently. Three infants in the full-term group and four in the preterm group were able to sit with arm support in a prop-sitting position.

Table 3 shows the median and range of SATCo scores for infants in all three conditions. Some infants achieved full trunk control, scoring a maximum of 7 in static and active conditions. Infants exhibited the lowest median and range of scores in the reactive condition, while the total scores show a wide range of values. Furthermore, the range of scores across the three conditions indicates that at least some infants exhibited segmental trunk control at the upper thoracic level.

Table 4 presents the median scores for routine daily activity. Three categories (A to C) indicate opportunities for upright activities in each dimension. Most scores were obtained during feeding, bathing, and diaper changes in Category A, as infants typically lie down. Bathing and dressing activities took less than 15 min to complete. Thus, the scores were low. Infants’ quiet play scores mainly fell into category A, as they were often placed to play in prone or supine positions in the cot. Some infants were scored in Category B when placed in prop-sitting or receiving sitting stimulation from caregivers, while independent sitters were in Category C. In active play, most scores were in Category A, as infants often lie down. Some infants who transitioned from sitting to crawling were in Category B, with no infants who played by climbing in Category C. Outings were not reported as infants were raised in the closed area, and sleeping was scored as zero.

**Figure 2:**
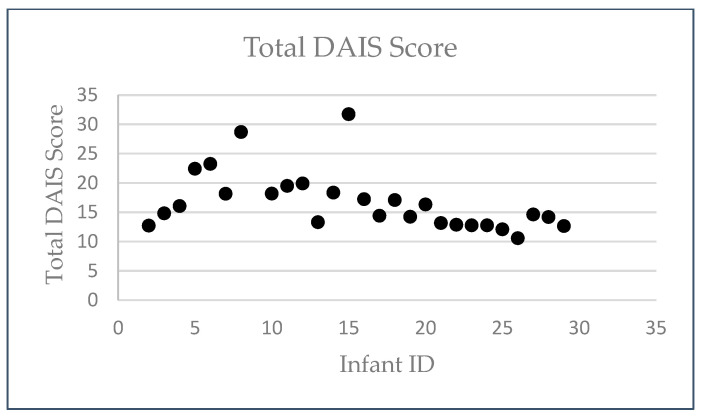
Distribution of Total DAIS Observational Blocks Across Infants.

The researcher plotted the Total DAIS Score for all infants in Figure 2 to illustrate the amount of observational data collected. Scores ranged from approximately 10 to 32, indicating broadly comparable observation coverage. Because the Total DAIS Score reflects the cumulative number of 15 min intervals (including proportional intervals for <15 min activities), this figure represents the variability in total scored observation blocks across infants (Figure 2).

### 3.2. The Difference in Segmental Trunk Control Between Full-Term and Preterm and/or Low-Birth-Weight Infants

Table 5 presents the differences in segmental trunk control between the full-term group and the preterm, and/or low-birth-weight group. There was a significant difference in SATCo scores between the two groups (*p* = 0.045; *p* < 0.05). In comparison, the SATCo scores across conditions showed no significant difference between the two groups (*p* > 0.05). The power of the test, calculated with G*Power (version 3.1), was 80.24% to detect the actual difference between the two groups. However, despite this statistically significant difference in the total SATCo score, the effect size was small-to-medium (r = 0.349), and the Bayesian evidence was only anecdotal (BF_10_ = 1.035), suggesting that the practical relevance of the difference should be interpreted cautiously.

**Figure 3:**
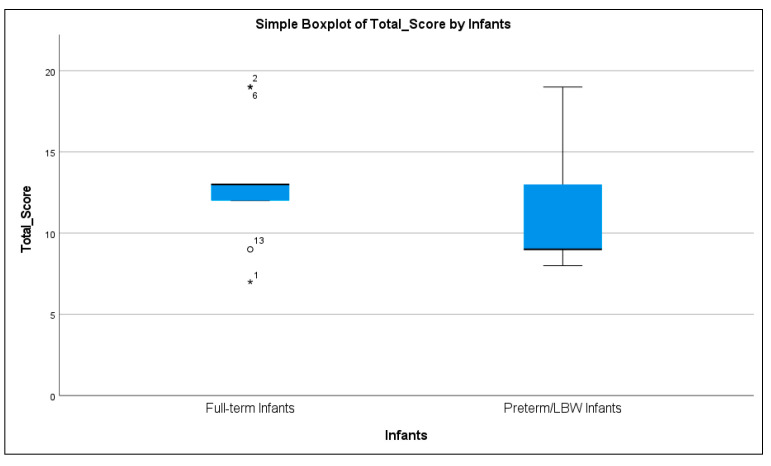
Distribution of Total SATCo Scores in Full-Term and Preterm/LBW Infants. Blue boxes show the score distribution (IQR) with the median line inside, whiskers indicate the normal range, circles mark outliers, and asterisks mark extreme outliers. Numbers in full-term infants are the infants’ ID.

Moreover, Figure 3 shows the distribution of total SATCo scores for both groups. Full-term infants had scores more closely grouped at higher values, whereas the preterm/LBW group showed a wider spread of generally lower scores. The figure also shows some overlap between groups. This visualization clearly shows that full-term infants tend to have higher total SATCo scores.

### 3.3. The Correlation Between Segmental Trunk Control in Sitting and Daily Activity of Infants in the Orphanage Setting

Significant correlations were identified between segmental trunk control in sitting and the daily activities of infants in the orphanage setting (Table 6). The relationship was found to range from moderate to good. Additionally, the data revealed a strong correlation between the active SATCo condition and DAIS scores, classified as good to excellent. Furthermore, the correlation between the static and reactive conditions of SATCo and the DAIS scores demonstrated a notable moderate-to-good correlation.

## 4. Discussion

Most infants in orphanages who cannot sit have limited opportunities for upright activities and exploring diverse environments. Thus, examining segmental trunk control in these infants is crucial. This study aimed to investigate the segmental trunk control during sitting and observe daily activities in the orphanage. The SATCo scores of full-term and preterm infants in an orphanage setting showed a significant difference between the groups, with the significance meeting the borderline of value. Full-term infants showed higher total SATCo scores than moderate-to-late preterm infants, implying that both biological maturity and environmental constraints jointly influence trunk control. Although the total SATCo score differed significantly between groups, the clinical significance of this difference should be interpreted with caution because the effect size was modest. The difference suggests that full-term infants may have slightly better upright sitting stability. Still, it does not reflect a shift from impaired to fully functional control, and both groups continued to show limited reactive control. Thus, this difference likely reflects minor variations in everyday motor performance.

This study found significant correlations between the total scores of SATCo and daily activity in each condition, ranging from moderate to good. A portion of the positive SATCo and DAIS correlation might reflect methodological overlap. SATCo measures trunk control in an upright sitting posture, while higher DAIS scores are given for activities that involve upright or active positions. Infants with better trunk control are therefore more likely to engage in these activities, which can elevate their DAIS scores and strengthen the observed correlation. However, our observations show that infants raised in the orphanage were more likely to exhibit reduced activity in an upright posture unless they could sit independently or were placed in supported seating. High scores in our observation were due to the extensive time spent in non-upright posture during daily activities. Our results suggest that the significant correlations further underscore the importance of opportunities to explore an antigravity posture during daily activities in supporting postural development, whether these infants were born full-term or premature.

Both groups of participants in our study had the same routine of child-rearing practices. Infants engaged in similar daily routine activities and positions, primarily remaining supine during feeding, bathing, and dressing, meaning that both groups experienced identical activities, mainly in category A. Additionally, infants had minimal exposure to varied play activities and experienced limited caregiver–infant interaction. As a result, they might have had fewer opportunities to experience external disturbances, which are essential for developing reactive trunk control. Even during stimulation sessions, the emphasis was often on basic sitting rather than on activities that cultivate reactive stability, resulting in reduced postural adaptability.

Due to the limited child-rearing practice in the orphanage, most activities occurred indoors or in the cots in a lying-down position. It is suggested that limited opportunities for upright positioning, particularly during play, may impact the development of segmental trunk control. This is confirmed by our results showing significant correlations between scores of segmental trunk control and daily activities across all conditions, with correlations ranging from moderate to good.

According to the SATCo score in each condition of all infants, the lowest score was obtained in the reactive condition. This study highlights that infants raised in orphanages may demonstrate limited development of segmental trunk control, particularly in reactive conditions. The SATCo scores of moderate-to-late preterm infants found in this study aligned with those of infants with a mean gestational age of 34.5 weeks and birth weight of 2206 g, as reported by Sangkarit et al. [3]. These authors noted that moderate-to-late preterm infants often lack complete segmental trunk control, especially in reactive conditions [3]. None of the 33 infants achieved a full score in the reactive condition, with a maximum stability score of 5. Although some were able to sit independently, they struggled to gain full trunk control in this condition. In contrast to Pin and colleagues’ study, both full-term and preterm groups in our study exhibited lower SATCo scores in each condition [25]. They reported median and range scores of 5 (4 to 6) in the static, active, and reactive conditions for 20 full-term infants aged 5 months. In addition, extremely preterm infants in their study with a mean gestational age of 27.2 weeks and a birth weight of 1989 g at a corrected age of 5 months had higher median scores: 5 (4 to 6) in the static, 5 (3 to 6) in the active, and 5 (3 to 5) in the reactive conditions [25]. Discrepancies in the results may arise from different child-rearing environments between the family and the orphanage.

The ability to sit independently is attributed to environmental contexts, such as movement experiences, the opportunity to sit on different surfaces, and time spent in an upright position [9]. Sangkarit’s study [3] showed that 55% of 35 premature infants began sitting independently around 7 months, highlighting the importance of reactive conditions for assessing sitting ability. Meanwhile, our study found that only 4 of 33 (12.1%) infants could sit independently. The previous study found that traditional mats and sleep mattresses had a positive impact on SATCo scores [11]. The softness of sleep mattresses can create instability, making it challenging for infants to maintain an upright posture. Transitioning from a firm to an unstable surface may challenge their movement [11]. Sitting on various surfaces, such as adult furniture, enhanced infants’ postural control, enabling them to refine their sitting skills [9]. Consequently, infants in the previous study who received more practice from their families were able to sit independently at 5 months of age [9].

Additionally, environmental interactions play a vital role in the development of trunk control. Motor development progresses through interactions between mothers and their children, as well as the children’s surroundings [11]. In this study, orphaned infants had limited interactions due to a low caregiver-to-infant ratio, receiving attention primarily during bathing, diaper changes, feeding, and brief periods of active play. Infants in the orphanage spent most of their time in the cot playing independently (quiet play). This aligns with Prommin et al. [26], who found that orphaned infants in confined cots lacked stimulation, were often bottle-fed, held by a pillow, and had limited caregiver interaction, mostly during quick diaper changes. Also, for safety reasons, these infants were raised in building conditions that significantly impacted infants’ physical, social, emotional, and overall development. Infants may not receive the necessary cues to adjust their posture in response to external stimuli, which hinders the development of reactive control. Limited experiences of sitting on various surfaces lead to a lack of necessary skills for maintaining balance and stability, which is crucial for reactive control stimulation.

Besides environmental factors in orphanages, factors that may be overlooked, like prenatal factors, such as maternal mental illness and substance use, can impact segmental trunk control. Though they had slightly different gestational ages, most infants in our study came from similar prenatal exposures and had near-identical family histories, e.g., born to mothers with drug or alcohol use and maternal mental illness. In this study, 43% of full-term and 11.8% of preterm infants had mothers with mental illness, while 31.2% of full-term and 29.4% of preterm infants were exposed to substance use. Cheng et al. [27] found that 38.3% of children born to substance-abusing mothers had developmental delays, including 17.2% with motor delays. Ross et al. [28] noted that substances like alcohol and nicotine can cross the placenta, harm fetal development, and disrupt neurogenesis and neurotransmitter systems essential for motor and behavioral growth.

Moreover, Gül, Gül, and Kara [29] identified maternal mental illnesses as one critical risk factor for developmental delays, particularly gross motor delays. Maternal stress can increase cortisol levels, which can disrupt fetal brain development and result in lower motor development scores, particularly during the third trimester [30]. A longitudinal study by Prommin et al. [19] also found that infants with low birth weight and maternal mental illness showed lower gross motor percentile variation.

Though we found a likely difference in SATCo scores between groups, the small-to-medium effect size and anecdotal Bayesian evidence indicate the lack of clinical difference in SATCo score, as the significance was at the borderline level. The final sample size was slightly below the calculated requirement, potentially increasing the risk of a Type II error in detecting small effects. This could explain the lack of pronounced differences across the SATCo components. This aligns with our observation that, despite a statistically significant difference and 80% test power, a lack of pronounced significant differences in SATCo scores may be due to the study’s limitations. The current study was conducted in a single orphanage, which limits the generalizability of our findings. The specific characteristics of this orphanage, including the caregivers’ practices and available support, may differ in other settings. In addition, most infants were born to mothers with similar prenatal exposure: mothers with risk factors like mental illness and alcohol or substance use; these factors cannot be overlooked. Most of the incoming infants into the orphanage come from parents with substance use issues, and they cannot take care of their babies. Limited daily activity in antigravity posture could be a factor affecting segmental trunk control, but the prenatal exposure also needs to be considered. Further research is required to identify both biological and environmental factors influencing trunk control in infants.

Our study contains several limitations. The cross-sectional design, however, limits the ability to track developmental progress to assess postural control in orphaned infants. A future longitudinal design study can also evaluate how caregiver interaction frequency and responsiveness affect postural control, highlighting practices that enhance motor development in low-engagement settings. This study was conducted in a single orphanage with homogenous caregiving routines. Future research should include a larger sample from multiple orphanages or a broader population to better understand environmental differences. Moreover, although this study conducted daily observations by a single researcher throughout the study, and the assessment is simple to use, not all DIAS assessment dimensions could be administered in the orphanage setting due to differences in child-rearing practices. Finally, we lack inter- and intra-rater reliability tests for the Daily Activity of Infant Scale (DAIS) assessment due to its observational nature, as the characteristics of different caregivers may influence reliability. Further study may use the video recordings to investigate the reliability test of the DAIS assessment.

## 5. Conclusions

This cross-sectional study found a statistical difference in SATCo scores between full-term and moderate-to-late preterm groups. Infants raised in the orphanage would exhibit delayed segmental trunk controls due to their prenatal exposure factors and limited child-rearing environment.

The study underscores the importance of improving the environmental context and child-rearing practices in the orphanage to support the development of trunk control, particularly in structured play or varied antigravity postures, through movement exploration, stimulation sessions, and high-quality caregiver interactions.

## Figures and Tables

**Table 1:** Characteristics of Infants (*n* = 33).

Demographics Data	Mean ± SD	
	Full-Term	Preterm/LBW ^#^
**Current age (months (days))**		
Chronological age	5 (13)	5 (22)
Corrected age	-	4 (25)
**Gestational age (week (day))**	38 (3)	35 (2)
Neonatal conditions		
Birth weight (g.)	2925.3 ± 206.7	2166.1 ± 328.5
Birth length (cm.)	46.8 ± 12.5	46.4 ± 2.9
Head circumference (cm.)	30.7 ± 8.2	30.1 ± 1.8
Apgar score		
1 min	8.8 ± 1.1	8.6 ± 0.7
5 min	9.4 ± 0.8	9.3 ± 0.5

^#^ LBW = low birth weight.

**Table 2:** Number of infants born to different maternal conditions during pregnancy.

Maternal Condition	Full-Term	Preterm/LBW ^#^
Mother with mental illness	7	2
Mother using drugs or alcohol	5	5
Child abandoned	-	2
Young mother	2	2
Postpartum complication	-	2
Mother using drugs + maternal infection + neonatal complication	-	1

**^#^** LBW = low birth weight.

**Table 3:** Score * of segmental trunk control of infants at the orphanage (*n* = 33).

SATCo Conditions	Median	Range
Static	5.0	3 to 7
Active	4.0	2 to 7
Reactive	3.0	1 to 5
Total scores	12.0	5 to 19

* Score of trunk control levels: 1 = head, 2 = upper thoracic, 3 = mid-thoracic, 4 = lower thoracic, 5 = upper lumbar, 6 = lower lumbar, 7 = full trunk control.

**Table 4:** Scores of daily routine activity of infants (*n* = 27).

DAIS Score	Median	Range
Feeding	3.6	1.9 to 5.6
Bathing	0	0 to 0.1
Dressing	0.2	0.1 to 2.7
Carrying	0	0 to 0.8
Quiet play	10.4	4.8 to 21.2
Active play	0.9	0 to 8.3
Total score DAIS	14.8	10.6 to 31.7

**Table 5:** The difference in segmental trunk control between the two groups.

SATCo Conditions	Median (Range)		Mann–Whitney U-Test U (*p*-Value)
	Full-Term (*n* = 16)	Preterm/LBW ^#^ (*n* = 17)	
Static	5.0 (3 to 7)	4.0 (3 to 7)	87.0 (0.061)
Active	4.0 (2 to 7)	4.0 (3 to 6)	101.0 (0.183)
Reactive	3.0 (1 to 5)	3.0 (2 to 5)	113.5 (0.384)
Total Score	13.0 (5 to 19)	10.0 (8 to 19)	81.5 (0.045)

^#^ LBW = low birth weight.

**Table 6:** Correlations between the SATCo and DAIS scores (*n* = 27).

SATCo Scores in Each Condition	DAIS Scores	
Static	0.734 **	(<0.001)
Active	0.805 **	(<0.001)
Reactive	0.723 **	(<0.001)
Total score	0.771 **	(<0.001)

** significant correlation (*p* < 0.01).

## Data Availability

Data generated or analyzed during this study are provided in full within the published article.
